# The Relationship Between Attachment to Pets and Mental Health and Wellbeing: A Systematic Review

**DOI:** 10.3390/ani15081143

**Published:** 2025-04-16

**Authors:** Katherine Northrope, Joanna Shnookal, Matthew B. Ruby, Tiffani J. Howell

**Affiliations:** 1 School of Psychology and Public Health, La Trobe University, Bendigo 3552, Australia; 2 School of Psychology and Public Health, La Trobe University, Melbourne 3086, Australiam.ruby@latrobe.edu.au (M.B.R.)

**Keywords:** dogs, cats, mental illness, pet–owner relationships, pet–owner bond

## Abstract

While pet ownership is sometimes associated with better mental health, research on this relationship has been inconsistent. Some researchers have considered what factors may impact this relationship, such as owners’ attachment to their pets. The aim of this systematic review was to investigate how attachment to pets relates to owner mental health and wellbeing. This review followed PRISMA guidelines. Searches were conducted using PsychInfo, Scopus and Google Scholar. Any peer-reviewed empirical studies, in English, investigating attachment to pets and mental health and wellbeing outcomes, were included. Of the 116 included studies, 15 studies found that higher attachment was associated with better mental health, 22 studies found that higher attachment was associated with worse mental health, 36 studies found mixed results and 33 studies found no significant relationship. Having a stronger attachment to one’s pet tended to be associated with better mental health when investigating outcomes in children, and when investigating outcomes related to wellbeing. Stronger attachment tended to be associated with worse mental health when investigating mental health symptomology (e.g., depression and anxiety). It may also be influenced by the owner’s relationships with other people. Due to the cross-sectional design used by studies in this review, we cannot infer the direction of causality for the relationship between attachment to pets and mental health. Results indicate that having a strong attachment to one’s pet is not necessarily associated with better mental health and wellbeing. Future longitudinal research is needed to better understand how attachment to pets affects mental health, and what factors may influence this relationship.

## Simple Summary

Pet ownership is sometimes, but not always, associated with better mental health, so it is necessary to consider factors influencing this relationship, such as the owner’s attachment to the pet. This systematic review examined 116 peer-reviewed studies that assessed the relationship between pet attachment and mental health and wellbeing outcomes. The results of these studies were mixed, with some finding that stronger attachment is associated with better mental health, some finding that it is associated with worse mental health while others found no relationship. Having a stronger attachment to one’s pet tended to be associated with better mental health when investigating outcomes in children, and when investigating outcomes related to wellbeing, compared to studies that investigated adults or outcomes related to mental health symptomology. The owners’ relationship with other people may also influence the relationship between their attachment to pets and mental health. However, as most studies were cross-sectional, we are unable to draw conclusions about the direction of causation of this relationship. Given that pet ownership is common, how this relationship may affect owners’ mental health and wellbeing and what factors may be influencing this has implications for the health of pet owners.

## 1. Introduction

Pet ownership is incredibly common. Approximately 69% of households in Australia own a pet [1], with dogs (48% of households) and cats (33% of households) the most commonly owned species. Similar rates of pet ownership have been reported in the USA [2]. The vast majority (85%) of pet owners surveyed in Australia said that their pets had a positive impact on their lives [1]. Pets are animals that live with humans in relatively long-lasting relationships and are typically associated with affection and companionship [3,4]. While popular media and anecdotal evidence suggests that the “pet effect” of owning a pet has positive outcomes for wellbeing [5], the research on this is less consistent [6,7].

Supporting claims that pets are beneficial for health, research from Australia, Germany and China found that pet owners make fewer doctor visits per year than non-owners [8,9,10]. Similarly, a 10-month prospective study that compared new cat and dog owners with non-owners found that both pet-owning groups had significantly improved physical and mental health outcomes during the first six months of ownership, whereas the non-owners reported no changes [11]. Pet owners have also reported higher life satisfaction and self-esteem than non-owners and have lower rates of loneliness and depression [12,13,14]. However, other research conflicts with these findings, with some studies finding that pet owners had higher rates of depression [15,16], and another study showing that getting a pet did not significantly change people’s loneliness [17].

To better understand the relationship between pet ownership and mental health, several systematic reviews have compared pet owners and non-owners. Reviews evaluating the relationship between pet ownership and physical and mental wellbeing [18,19], and loneliness [20], have found that some studies suggest pet ownership is associated with better mental health and wellbeing, whereas others find it is associated with worse mental health and wellbeing, and yet others find no differences between pet owners and non-owners. Similar results have been found when focusing on specific populations, like the elderly [21]. One systematic review that investigated both quantitative and qualitative studies on the impact of pet ownership on mental health symptoms for those with a diagnosed mental health condition again found mixed results [22]. However, their review of the qualitative research found that the most pet owners saw their pets as helping their psychological health by providing comfort, social interaction and a sense of self-worth. Some negative impacts noted by owners included financial costs and grief over the death of their pets. These reviews provide a complicated picture of pet ownership, suggesting that pet ownership by itself is not a clear predictor of mental health outcomes, and that other factors may impact this relationship.

One explanation for these mixed findings is that most research focuses on pet ownership, per se, rather than the quality of the relationship, or the attachment to the pet [19,23]. While this attachment system is most evident in infancy and early childhood for primary caregivers, it is active in other relationships across the lifespan [24]. There are three main styles of attachment—anxious, avoidant and secure—although terminology may vary depending on how these are being measured and some researchers also consider additional styles such as disorganised or unclassifiable [24,25]. In adulthood, those who are higher in attachment-related avoidance tend to avoid closeness in relationships and avoid becoming too dependent on others, whereas those who are higher in attachment-related anxiety desire closeness and may become distressed if they think their attachment figure is not readily available [24]. These two styles are classified as being insecure. Those who are low on both dimensions are classified as being secure in their attachment style, as they feel comfortable being close with other people and do not worry about being abandoned by others. Insecure attachment styles in human relationships have consistently been associated with negative mental health outcomes [26].

Some researchers have suggested that pets may function as an attachment figure [27]. In one study, 14% of owners reported that their pet was their primary attachment figure [28]. Other research using modified versions of human attachment measures found that participants reported more secure attachments with their pets than with their partners [29]. One of the early discussions of applying attachment theory to pets suggested that owners who have developed a basic distrust of human attachment may instead form an intense attachment to their pets [30]. One of the systematic reviews previously discussed also reported on 13 studies that measured attachment to pets and how this related to mental health outcomes [18]. The results of this relationship were also mixed, with five studies finding attachment was associated with better mental health, two studies finding it was associated with worse mental health, four finding mixed outcomes and two finding no relationship at all with mental health. However, this review [18] investigated mental health outcomes comparing pet owners and non-owners more generally, and did not specifically search for studies focusing on the attachment relationship between the owner and the pet, so may thus have missed relevant studies as part of their review. More recently, a systematic review of the relationship between attachment to pets and levels of depression found that in most studies, higher levels of attachment were associated with higher levels of depression [31]. Similarly, this review [31] specifically focused on outcomes related to depression, and did not report the results of the relationship between attachment to pets and other mental health outcomes.

Many scales aim to measure pet–owner relationship quality [32]; however, they typically assess the strength of the owner’s bond with the pet, rather than secure and insecure attachment styles as is typical in human relationship research [33]. One notable exception to this is the Pet Attachment Questionnaire (PAQ), which assesses self-reported attachment orientations in the human–pet relationship on two factors—anxiety and avoidance [34]. The most commonly used scale to study the human–animal bond [32] is the Lexington Attachment to Pets Scale (LAPS) [35], which measures the strength of the attachment to one’s pet rather than attachment style. Other commonly used scales measure similar aspects related to emotional closeness with the pet, but also ask about specific types of interactions between owners and their pets [36]. This may be problematic when trying to compare attachment between different species, as some interactions (e.g., playing, exercising, travelling with) may be more common for pets such as dogs, but less common for pets such as cats or small rodents [36,37]. Some scales, such as the Monash Dog Owner Relationship Scale (MDORS) [38] and the Cat Owner Relationship Scale (CORS) [37], measure emotional closeness with the pet, but also measure interactions that may be specific to that species of pet.

While psychiatric research has traditionally focused more on mental illness and specific disorders (e.g., depression, anxiety) [39], broader definitions of mental health not only take into consideration the presence or absence of mental illness symptoms, but also consider aspects of wellbeing [40]. Rather than viewing mental health as existing on a continuum, from experiencing severe mental illness to being completely mentally healthy, mental illness and mental health are viewed as two correlated but distinct axes [41]. Mental health is not only the absence of mental illness, but also incorporates aspects of wellbeing (e.g., how a person perceives the overall quality of their life) [40]. More importantly, research suggests that the genetic and environmental factors that predict mental illness differ from those that predict mental wellbeing [42]. Pet ownership may affect specific aspects of mental health and quality of life in different ways, so when exploring the relationship between attachment and mental health, it is useful to explore a broad range of outcomes [43].

In summary, while several systematic reviews have investigated the relationship of pet ownership with mental health, and a recent review has investigated the relationship between attachment and depression, at present there is no clear consensus on how attachment to pets affects a range of mental health and wellbeing outcomes. This systematic review aims to address the research question of how attachment to pets is related to owner mental health and wellbeing. It includes both studies that measure attachment strength, and studies that measure attachment style.

## 2. Materials and Methods

This review was conducted in accordance with the Preferred Reporting Items for Systematic Reviews and Meta-Analyses (PRISMA) statement [44].

### 2.1. Eligibility Criteria

This review included peer-reviewed journal articles, chapters, theses, and dissertations containing empirical studies that evaluated both attachment to one’s pet and any mental health and wellbeing outcomes. We only included manuscripts written in English. Grey literature like chapters and theses were included based on recommendations to include these in systematic reviews in an attempt to account for publication bias [45]. We excluded studies that only focused on physical health outcomes or included animals that are not the participants’ current pet, such as animal-assisted therapy or assistance animals, or studies focusing on previously owned pets. No exclusions were made based on study design.

### 2.2. Literature Search Process

An initial literature review was conducted to determine appropriate terms used in the research that could identify relevant studies and were narrowed down through discussion with the authorship team. The final terms used were also discussed with a La Trobe University librarian to ensure they were appropriate for the databases used in this review. We entered the following search terms into Scopus and PsychInfo databases: pet* OR companion animal* OR cat* OR dog* AND attach* AND mental health OR mental illness OR wellbeing OR depress* OR anx*. An additional search using the same terms was completed using Google Scholar. For Google Scholar, we reviewed the titles of the first 200 articles, as recommended in previous research [46,47]. Studies identified using this method were first downloaded into Endnote and then transferred to Covidence to assist with the review process. This was initially completed in April 2024 and was rechecked using the same search strategies to include all studies up until 30 November 2024. Studies identified while reading the papers that were already deemed eligible for inclusion were also checked to see if they met inclusion criteria. These studies were also downloaded into Endnote and then transferred to Covidence.

### 2.3. Data Extraction

The first reviewer (KN) completed the title and abstract screening in full. The second reviewer (JN) checked all titles excluded at this stage and flagged any studies that appeared to meet inclusion criteria [48]. Any disagreements at this stage were resolved by discussion. Both reviewers then independently completed the full-text screening as is recommended for systematic reviews to avoid errors and risk of bias and to ensure all relevant studies are included [45,48,49]. Again, any disagreements were resolved by discussion. For each study, sample size, gender, country, pet species and other relevant population characteristics are reported. We also report the scale used to measure attachment, what mental health outcome was measured, and what scale was used to do this, and the relationship between attachment and mental health.

### 2.4. Quality Analysis

The first author evaluated the quality of the included studies using a checklist designed by the Joanna Briggs Institute (JBI) for Analytical Cross-Sectional Studies [50]. The JBI checklist evaluates quality of study design by asking about inclusion criteria, reliability of the exposure and outcome measures and statistical analyses via eight closed-ended questions, with responses of yes, no or unclear. One of the questions asks if the study subjects were described in detail. For this, we required that the studies provided demographics for age and gender, as well as the pet type/s investigated. For the question asking if the exposure was measured in a valid and reliable way, we checked whether the measure of attachment to pets had been validated (either in the study using it or previous research) and shown to be internally reliable. For outcome measures used to assess mental health and/or wellbeing, we also checked that these had been validated and shown to be internally reliable. Some studies included only a single-item measure, but the authors indicated that this had been validated by previous research. Where we could not find any previous research that provided reliability statistics or other evidence that the measure had been validated, we answered unclear for the study for that question. The checklist also includes a “not applicable” option for questions, as not all questions are relevant for all study designs. For the studies included in this review, questions four, five and six were not applicable. Question four asked whether there was an objective, standard criteria used for measurement of the condition, which was not applicable as we are reporting on the relationship between attachment and mental health, rather than comparing conditions. Questions five and six asked about confounding factors, and how this may have affected group comparisons, which again was not relevant in the context of the relationship we are reporting on.

## 3. Results

**Figure 1:**
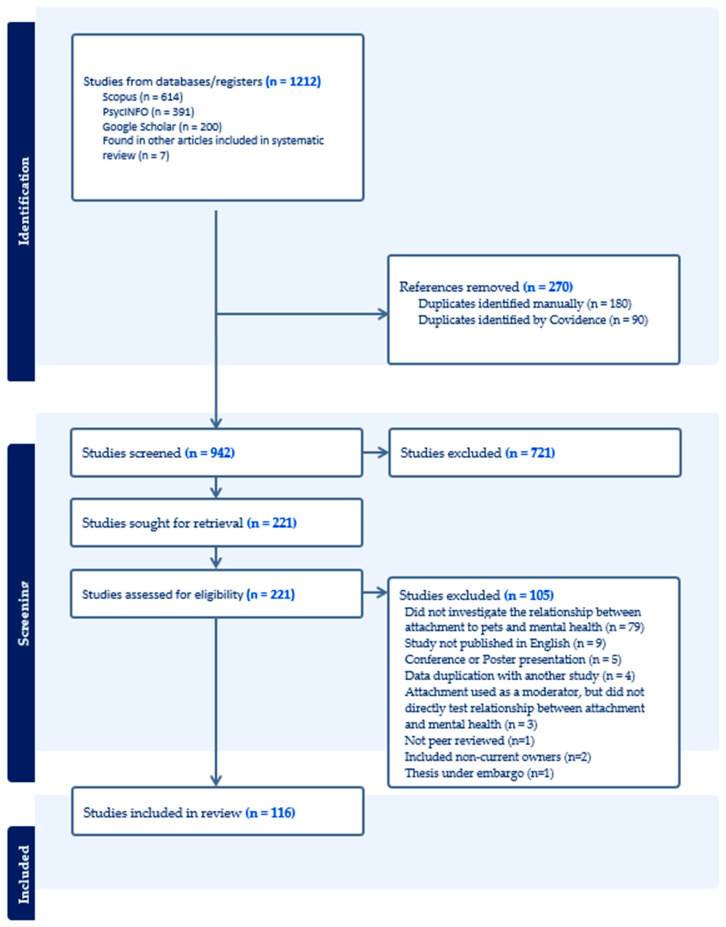
Article selection process detailing the number of articles included and excluded at each step of the review using the PRISMA flow guidelines.

The initial search identified 1212 articles. After removing 270 duplicates, titles and abstracts were screened for 942 studies by the first author, which lead to 721 studies being excluded as irrelevant. The second author reviewed these 721 studies and returned any studies that looked potentially relevant, based on the title and abstract, for a second review. This resulted in 206 studies being included for full text review, which was completed by the first two authors. After this screening process, 106 studies were included in the final review. Seven additional studies that met inclusion criteria were identified when reading the text of these 106 studies. Three additional studies were also identified in November 2024, resulting in a final sample of 116 studies. The PRISMA flow diagram for this search strategy is presented in Figure 1. The most common reason for not including a paper in the systematic review was that it did not explore the relationship between attachment to pets and mental health—e.g., one study measured participants’ attachment to pets and mental health outcomes, but used these measures to compare owners and non-owners, rather that the relationship between the two variables [51].

Studies were published between 1983–2024. Of these 116 studies, 86 were journal articles, 27 were theses, and 3 were book chapters. While these studies were published in a range of countries, the overwhelming majority used samples from the USA. The majority of studies included in this review were cross-sectional studies, with the exception of two cohort studies [52,53]. Neither of these studies found that attachment to pets was associated with mental health.

Attachment to pets was measured using a range of different scales, but the LAPS [35] was the most commonly used scale. There were also a range of different mental health and wellbeing outcomes. Abbreviations used for both attachment and mental health outcomes are described in Table 1.

The full results are presented in Table 2. Where possible, we report the mean or median for age of participants in each study sample. Some studies did not provide this information, in which case we report the age based on the available information from the original paper. Where studies have used both a measure of attachment strength (e.g., LAPS) and attachment style (e.g., PAQ), results are presented separately in the appropriate section. 

Of the 116 studies, 15 studies found that higher attachment was associated with better mental health, 22 studies found that higher attachment was associated with worse mental health, 36 studies found mixed results and 33 studies found no significant relationship between attachment to pets and mental health and wellbeing outcomes. Of the 36 studies that found mixed results, 22 studies found that stronger attachment to pets was associated with worse mental health and wellbeing outcomes for some of the outcomes used in their study, but with no relationship between attachment and some of the other outcomes. Ten of the mixed studies found that stronger attachment to pets was associated with some better mental health and wellbeing outcomes, but that there was no relationship between attachment and some of the other outcomes measured in the study. The last two mixed studies found that attachment was associated with both better and worse mental health outcomes. Finally, 14 studies measured avoidant and anxious pet attachment and how this relates to mental health and wellbeing.

For the studies that found that higher attachment was associated with worse mental health, higher attachment was associated higher levels of depression [155,199,201,207,211,214,220,233,241], dissociation [196,197,198], loneliness [202,205,206,207,216,220,241], PTSD and burnout [208], anxiety [201,214,220,232,240], separation anxiety [200], stress [205,213,214,225,231,243] and worse general mental health [23,208,209,210,212,217], and lower wellbeing [203,215], quality of life [239] and resilience [208].

For the studies that found that higher attachment to pets was associated with better mental health and wellbeing outcomes, higher attachment was associated with lower levels of depression [128,158], stress [139,157], loneliness [126,129], anxiety [158] and suicide risk [127], and higher life satisfaction [133,147], happiness [130,137], positive mood [133], wellbeing [60,129,136,144,151], emotional regulation [129] and better mood [152].

For the 36 studies that found no relationship between attachment and mental health, attachment to pets was not associated with levels of stress [161,163,167,172,173,174,184,189,230], depression [159,162,165,171,174,175,179,182,189,193], dissociation [194], general mental health [52,178,181], mood states [188], happiness [251], quality of life [43,248], life satisfaction [251], loneliness [166,169,170,176,185,195], anxiety [182], emotional exhaustion [189] or wellbeing [182,186,254].

For the studies that found that attachment was associated with worse mental health for some variables but not others, in some cases, this refers to attachment being associated with only certain aspects of mental health (e.g., higher attachment was associated with higher anxiety, but not associated with depression) [232]. In other cases, some papers reporting results of multiple studies found that attachment was only significantly related to poorer mental health outcomes in some, but not all of the studies presented (e.g., [235]). Others found that higher attachment was associated with worse mental health only for certain groups (e.g., those aged 35–44) [148]. For measures of attachment that had multiple subscales (e.g., the LAPS), only some of these subscales were significantly associated with poorer mental health [59,221,222,224,228,234,235].

Similarly, for the studies that found attachment was associated with worse mental health for some variables but not others, in most cases, attachment was associated with only certain aspects of mental health—e.g., higher attachment was associated with lower depression and anxiety, but not phobic anxiety [158]. For measures of attachment with multiple subscales (e.g., the PALS), only some of these subscales were significantly associated with wellbeing [154]. For one of the studies looking at different age groups of children, higher levels of attachment were associated with higher self-esteem for the total sample and high schoolers, but there was no significant relationship for elementary and middle schoolers [156].

For the two studies that found that higher attachment was associated with both better and worse mental health/wellbeing outcomes, higher attachment was associated with higher depression, anxiety and loneliness, but was unrelated to negative affect in one study [244], and higher negative affect but not loneliness in the other study [245]. Both of these studies also found that higher attachment was associated with positive affect as measured by the PANAS [117].

Finally, 14 studies measured avoidant and anxious pet attachment. The PAQ [34] was used for 13 of these studies, with the other study using a modified version of the ECR [255]. Having an anxious attachment style towards one’s pet was associated with poorer outcomes in 11 of the studies, including higher levels of mental health symptoms [34,212,246], neuroticism factors (e.g., anger, anxiety, depression [238]), negative affect [249] and suicide risk [127], and lower quality of life [250], wellbeing [247,249] and positive affect [249]. Having a more avoidant attachment style towards one’s pet was less consistently associated with mental health, with only five of the studies finding a significant relationship. A more avoidant attachment style was associated with higher levels of mental distress [212] and stress [213], and with lower positive affect [249] and wellbeing [247,253]. Three of the studies found no relationship between attachment style and mental health outcomes.

### Quality Analysis

The quality of the studies included in this review was generally good, according to the JBI criteria, with 70.09% of these studies meeting the criteria to be classified as “yes” to all five questions. The results of these are presented in Table 3. There were also issues that are not included in the JBI criteria. For instance, some studies included in this review had very small samples, and most studies in this review relied on convenience samples, which typically resulted in women being overrepresented. Further, as participants self-selected to take part in these studies, samples might not be representative of the general pet-owning population in some ways; participants may be more attached to their pets than the average owner. Indeed, several studies noted ceiling effects for attachment measures [133,165,171,201]. Finally, as all studies included in this review were cross-sectional, we cannot determine a causal relationship between the examined variables.

## 4. Discussion

The purpose of this review was to explore the relationship between attachment to pets and mental health and wellbeing. The search strategy identified 116 studies that met our inclusion criteria. Of these 116, 15 studies found that higher attachment was associated with better mental health, 22 studies found that higher attachment was associated with worse mental health, 36 studies found mixed results and 33 studies found no significant relationship between attachment to pets and mental health and/or wellbeing outcomes. An additional 14 studies investigated attachment style, as opposed to strength, and mental health outcomes. Given the differences in how attachment and mental health were measured, and the differences in sample characteristics, it is difficult to directly compare results across all the studies discussed in this review. However, some general trends in relationships emerged.

There appeared to be some differences in the relationship between attachment to pets and outcome measures, depending on how mental health and wellbeing were measured. This was particularly the case when investigating aspects of wellbeing, as 17 of the 25 studies that found higher attachment associated with better outcomes measured wellbeing, quality of life or happiness. Higher attachment to pets was typically associated with worse outcomes in studies that measured mental health symptomology (e.g., depression, anxiety). For example, three of the four studies that investigated levels of attachment and dissociative symptoms also found that higher attachment was associated with worse mental health, with the other study finding no relationship between the variables. The results for the relationship between attachment and stress were mixed, although most studies found no relationship between these variables. The relationship between attachment and loneliness was also mixed. The UCLA Loneliness scale was the most commonly used scale to measure loneliness, which may not be an appropriate measure of loneliness for human–pet outcomes, as it focuses on loneliness due to a lack of human interaction [245]. Given the cross-sectional nature of these studies, we cannot draw any conclusions about causation based on these results. While having a strong bond with one’s pet may have benefits when it comes to wellbeing, this bond is likely not able to reduce or prevent symptoms related to mental health disorders, which may require treatment by trained medical professionals. Those experiencing mental distress may also be more likely to form a stronger relationship with their pet as a way of trying to manage or distract from their symptoms.

The relationship between attachment to pets and mental health sometimes varied depending on age group. Nine of the twenty-five studies that found that higher attachment was associated with better mental health specifically focused on children and adolescents [60,126,129,130,136,142,151,153,156]. In fact, no studies found that higher attachment to pets in children was associated with worse mental health, although three studies found no relationship between attachment to mental health during adolescence. Several studies did find that younger pet owners had higher levels of attachment [60,151,178,218]. Higher attachment was typically associated with worse mental health from university age onwards.

Of the 12 studies that found that higher attachment was associated with better mental health for at least some outcomes in the general adult population, 8 of these used samples from Asian countries [139,143,144,146,147,150,152,157]. As most of the studies included in this review were based in Western countries, we were unable to draw any definitive conclusions, but future research is needed to examine whether there are cultural differences in how pet attachment relates to mental health. Results for the elderly were also mixed, with 6 out of 14 studies focusing on this this age group finding that higher attachment to pets was associated with worse mental health, 6 finding no relationship, and 2 finding that higher attachment was associated with better mental health.

Higher attachment was typically associated with worse mental health in studies that took place during the height of the COVID-19 pandemic, with 11 of 14 studies undertaken during this time drawing this conclusion [59,210,214,216,221,222,225,234,237,241,245]; one study found that higher attachment was associated with better mental health [144], and one study found no relationship [189]. Our results are consistent with a scoping review of studies on pet ownership and wellbeing outcomes more generally during the pandemic, which found mixed results [256]. Their scoping review also considered the relationship between attachment to pets and both physical and mental health and found a more balanced spread of outcomes between attachment and health in terms of better/worse/neutral outcomes than the current review.

Finally, 14 of the included studies investigated style of attachment to pets rather than the more commonly used scales that measure pet attachment in terms of strength. The PAQ [34] was used for 13 of these studies, with the other study using a modified version of a scale used to measure attachment style in human relationships, the ECR [255]. In 11 of these studies, having an anxious attachment style towards one’s pet was associated with poorer mental health, whereas having an avoidant attachment style was only associated with poorer mental health in 5 of the studies. This is consistent with research relating to attachment styles in human relationships, as while both anxious and avoidant attachment styles are associated with poorer mental health [26], this relationship is more consistently reported for those with anxious attachment styles [257,258]. In human relationships, those with avoidant attachment styles may be able to cope with day-to-day life stress but find that they are unable to cope with more extreme forms of stress like divorce or illness where support from other people may be necessary [259]. In comparison, those with anxious attachment styles become distressed when they feel like their attachment figure is not readily available, and are more likely to ruminate or behave in self-defeating ways, which further contributes to their risk of poor mental health [26,259].

While only 14 studies in this review measured pet attachment in terms of anxious or avoidant style, anxious attachment was more consistently associated with poorer mental health and wellbeing compared to avoidant attachment. For those interested in how pet ownership (and particularly attachment) relates to mental health, the PAQ may be a valuable tool to understanding this relationship. Future research can also help establish what factors are associated with having an insecure attachment with one’s pet. For example, some research has found that having an insecure attachment style in human relationships is associated with having an insecure attachment style [34,212], and those who score higher on neuroticism also tended to be more anxiously attached to their pets [34,253], which are both factors associated with poorer mental health [26,260].

Given that the overall relationship between attachment to pets and mental health was so mixed, it may be worth considering what other factors may be influencing this relationship, such as pet type. Most studies included in this review focused on cat and dog owners. Where studies did include other pet types (e.g., birds, small rodents), they made up such a small minority of the sample that the authors were unable to compare outcomes based on species type for the “other” category. One study did examine birds specifically, finding that higher attachment was associated with higher levels of loneliness [216]. A small number of studies did include horse owners, with one study finding similar outcomes for attachment compared to other pet owners [154], while three other studies reported that horse owners reported higher levels of attachment compared to other pet types [133,179,245]. One study also noted that horse owners had higher levels of positive affect compared to owners of other types of pets [245]. Due to the relatively small number of studies investigating pet types other than cats and dogs, we are unable to draw any meaningful conclusions about species differences for attachment and mental health.

In the studies reviewed in this paper that focused on cat and dog owners, dog owners typically scored higher on attachment to their pets [52,151,152,157,176,219,231,241,245]. Other research found no difference for cat and dog owners in levels of attachment towards their pet [195]. It is unclear whether these differences in levels of attachment are due to certain scales being more suitable for dogs because they asked questions about frequency of behavioural interactions that are more common for dogs than other species—e.g., exercising and playing [36]—or whether there are true differences in the way that owners bond with different pet species. Cat owners also reported more avoidant attachment style towards their pets compared to dog owners [34,253]. This may be due to species differences in typical behaviour, with cats stereotypically being more independent than dogs, although it is unclear if those with an avoidant attachment style are more motivated to seek out a cat, or whether the cat’s behaviour leads to a more avoidant attachment style [34].

In one study, dog owners with higher attachment had better mental health outcomes [231]. Several studies investigating pet ownership more generally have found that dog owners tend to have better mental health than owners of other species [12,261,262]. This may be due to dog owners getting more exercise through walking their pets, which also provides an opportunity for socialisation with other members of the community [14]. Other evidence suggests that dog and cat owners may differ in terms of personality—e.g., cat owners may experience poorer wellbeing due to being higher on neuroticism compared to dog owners [12]. Most studies included in this review considered cat and dog owners together as “pet owners” to explore the relationship between attachment to pets and mental health. Given that there may be some species differences in terms of both level and style of attachment, it will be useful for future researchers to explore the relationship with attachment and mental health separately for different species of pets.

Women typically scored higher than men on attachment to their pets [43,52,60,126,128,151,152,156,179,185,212,218,235]. This is consistent with a previous review on gender differences in human–animal interactions, which found that women tended to be more attached to their pets than men, although the effect sizes for this were small [263]. One exception in this review was Smith [264], which investigated attachment to pet dogs amongst married couples, finding that husbands reported higher attachment to their dogs than their wives. Several other studies found no differences between men and women in terms of attachment [172,250]. Other research, however, found that men were more likely than women to have an avoidant attachment style towards their pets [212,252]. One study also found that higher attachment was associated with worse mental health for women, but there was no significant relationship for men [212]. This is consistent with previous research which found that women with pets had higher rates of anxiety than non-owners, whereas men with pets had lower rates than non-owners [176]. Other research during the COVID-19 pandemic found that pet ownership was associated with lower wellbeing for women, but not men [261]. Similar to the species differences discussed above, it may be useful for future researchers to explore the relationship with attachment and mental health separately for men and women, particularly given that many studies in this review had samples consisting primarily of women.

As many studies in our review suggest that higher attachment to pets is associated with worse mental health, it is important to consider why this is the case. One common explanation for this finding was that higher attachment to one’s pet may be driven by some other factor, such as the quality of the owner’s relationships with other people [209]. For example, several studies found that those with insecure attachment styles in their human relationships tended to have a stronger attachment to their pets [199,209,212]. Indeed, two of these studies tested mediation effects for attachment style to other humans and found that including this caused the relationship between attachment to dogs and mental health to become non-significant [209,212]. Several studies also found that those who were more highly attached to their pets reported less social support [203], higher self-reliance [181] and a smaller social network, and typically lived alone [245]. Other studies included in this paper that considered levels of social support from other people as well as attachment to pets found that social support from other people was a better predictor of better mental health [52,128,149,150]. In one study that found no relationship between pet attachment and mental health, an insecure attachment style to other humans was associated with higher stress levels [164]. It may also be that those who have higher levels of distress may rely more on their pet for comfort [240,245]. Again, this may particularly be the case for owners living alone [245], or with a reduced social network [219]. Not all research has found that a having a close relationship with a pet is associated with having poor human relationships, with some studies finding that pets can be an additional form of social support above and beyond that received from other humans [13,28], and one finding that higher attachment to pets was associated with higher levels of social support from humans [185]. As such, more research is needed for whether pets substitute or complement human relationships, and how this may affect mental health outcomes for these owners [211].

### Limitations

There are several limitations that are important to consider for this systematic review. Firstly, while we aimed to include all relevant literature on the relationship between attachment to pets and mental health, we cannot be sure that our search strategy identified every study that would have met our inclusion criteria. In particular, we note that only the first 200 results identified using our search strategy through Google Scholar were screened to see if they met our inclusion criteria. Given that our search terms resulted in over 35,000 results, we made the decision to only include the first 200 results based on recommendations by previous researchers [46,47] but acknowledge that some relevant studies may have been missed using this strategy.

Secondly, as noted in the overall quality assessment, the majority of studies relied on convenience samples, which resulted in samples that may not be representative of the general pet-owning population. Participants who self-selected to take part in these studies may have been motivated to do so because of especially high interest in their pets, and may have scored higher on the measures of attachment than the average pet owner. Similar to other human–animal research, women made up the majority of participants in most studies included in this review. Most studies included in this review also used samples from Western countries. This means that the results of these studies may not generalise to the average pet owner, particular men and people in non-Western contexts.

Thirdly, while the authors of many of the included studies discussed attachment to pets in relation to previous research on attachment theory in human relationships, most attachment scales used in human–animal research do not measure attachment in the way it has been conceptualised in human–human research [33]. The great variation in how attachment was defined and measured makes it difficult to interpret and compare outcomes across studies [240]. Some of the scales included items that assessed the quality of the relationship between the owner and their pet, whereas some items used were less clearly relevant. For example, the Pet Relationship Scale (PRS) [265] was used in several studies as a measure of attachment, but the original authors designed this scale as a measure of owners’ relationship and attitude to their pets more generally, and included items about sharing food with pets, which is not clearly related to attachment. Some scales used may also not be appropriate for all species of pets [36].

Finally, as all the studies included in this review are cross-sectional, we are unable to infer the direction of causation between attachment to pets and mental health and wellbeing. While it is possible that the relationship with one’s pet could directly lead to better or worse outcomes, it is also possible that those experiencing either high levels of wellbeing or mental distress may be more motivated to seek out their pet to share their emotional experience with or seek emotional support from due to their emotional state. There may also be other factors that impacting both attachment and mental health outcomes, such as the owners’ personality or relationship with other people. Given that it is difficult to interpret the cause-and-effect relationship of attachment to pets and mental health outcomes, longitudinal research may help with understanding how these variables may affect each other over time.

## 5. Conclusions

This review found mixed evidence for how attachment to pets relates to mental health and wellbeing. Having a stronger attachment to one’s pet was associated with better outcomes in children when investigating outcomes related to wellbeing. Stronger attachment was more often associated with worse mental health when investigating outcomes related to mental health symptomology (e.g., depression and anxiety). Many studies found no relationship between attachment to pets and mental health, suggesting that while attachment may important, there are likely other factors that impact pet owners’ mental health. Factors that may influence this relationship include gender of the owner and the pet species, so future research may benefit from investigating these as potential moderators. The quality of the owners’ relationship with other people may also influence both their attachment to their pets and their mental health. A small number of studies investigated levels of avoidant or anxious attachment to pets (i.e., using the PAQ [34]), and found that having an anxious attachment to one’s pet was associated with worse mental health. This pattern of results was more consistent than those studies that measured attachment strength or a more general bond. Future researchers who are interested in using attachment theory to understand people’s relationship with their pets should ensure that the measures they use reflect attachment theory rather than assessing a more general sense of a strong bond. Given that pet ownership is reasonably common, understanding how this relationship may affect pet owners’ mental health and wellbeing and what factors may be influencing this relationship has implications for people worldwide.

## Figures and Tables

**Table 1:** Abbreviated scale names for attachment and mental health outcomes included in final sample of systematic review.

Outcome	Abbreviation	Measure/s	Reference
Attachment	C/DORS	Cat/Dog-Owner Relationship Scale	Howell et al. [37]
	CCAS	Comfort from Companion Animals Scale	Zasloff [36]
	CABS	Companion Animal Bonding Scale	Poresky et al. [54]
	DAQ	Dog Attachment Questionnaire	Archer and Ireland [55]
	CENSHARE PAS	The CENSHARE Pet Attachment Survey	Holcomb et al. [56]
	LAPS	Lexington Attachment to Pets Scale	Johnson et al. [35]
	MDORS	Monash Dog-Owner Relationship Scale	Dwyer et al. [38]
	OPRS	Owner-Pet Relationship Scale	Winefield et al. [52]
	PALS	Pet Attachment and Life Impact Scale	Cromer and Barlow [53]
	PAS (1988)	Pet Attachment Scale	Albert and Bulcroft [57]
	PAS (1996)	Pet Attachment Scale	Staats et al. [58]
	PAQ	Pet Attachment Questionnaire	Zilcha-Mano et al. [34]
	POCS	Pet Owner Connectedness Scale	Oliva and Johnston [59]
	SAPS	The Short Attachment to Pets Scale	Marsa-Sambola et al. [60]
General Mental Health	BSI	Brief Symptom Inventory	Derogatis and Melisaratos [61]
	BSI-18	Brief Symptom Inventory-18	Derogatis [62]
	GHQ-12	General Health Questionnaire 12	Goldberg et al. [63]
	MHI	Mental Health Inventory	Veit and Ware [64]
	SF-12	Short-Form-12 Health Survey	Ware et al. [65]
	SF-36	Short-Form-36 Health Survey	Ware [66]
Anxiety	BAI	Burns Anxiety Inventory	Burns [67]
	CCI	Crown Crisp Experiential Index	Crown and Crisp [68]
	GAD-2	Generalized Anxiety Disorder-2 scale	Sapra et al. [69]
	GAD-7	Generalized Anxiety Disorder-7 scale	Spitzer et al. [70]
	GAS-10	Geriatric Anxiety Scale	Carlucci et al. [71]
	LSAS	The Liebowitz Social Anxiety Scale	Liebowitz [72]
	SA-10	Severity Measure for Separation Anxiety Disorder–Adult	Craske et al. [73]
	STAI	State Trait Anxiety Inventory	Spielberger et al. [74]
Depression	BDI	Beck Depression Inventory	Beck et al. [75]
	BDI-II	Beck Depression Inventory-II	Beck et al. [75]
	CES-D	The Center for Epidemiological Studies Depression Scale	Radloff [76]
	CES-D-10	The Center for Epidemiological Studies Depression Scale 10	Andresen et al. [77]
	DASS	Depression Anxiety and Stress Scale	Lovibond and Lovibond [78]
	DASS-21	Depression Anxiety and Stress Scale-21	Lovibond and Lovibond [56]
	GDS	Geriatric Depression Scale	Yesavage et al. [79]
	K6	Kessler Psychological Distress Scale	Kessler et al. [80]
	PHQ-2	Patient Health Questionnaire-2	Thombs et al. [81]
	PHQ-9	Patient Health Questionnaire-9	Kroenke et al. [82]
Stress	CSSQ	COVID-19 Student Stress Questionnaire	Zurlo et al. [83]
	Parental SS	Parental Stress Scale	Berry and Jones [84]
	PSQ	Perceived Stress Questionnaire	Levenstein et al. [85]
	PSS	Perceived Stress Scale	Cohen et al. [86]
	PSS-10	Perceived Stress Scale-10	Cohen and Janicki-Deverts [87]
	PSI-SF	Parenting Stress Indexed Short Form	Abidin et al. [88]
Dissociation	DES	The Dissociation Experiences Scale	Bernstein and Putnam [89]
Loneliness	ABLS	Abbreviated Loneliness Questionnaire	Ellison and Paloutzian [90]
	DLS	The Differential Loneliness Scale	Schmidt and Sermat [91]
	SELSA-S	Social and Emotional Loneliness Scale for Adults	DiTommaso et al. [92]
	UCLA-LS	UCLA Loneliness Scale	Russell et al. [93]
Happiness	OHQ	Oxford Happiness Questionnaire	Hills and Argyle [94]
	SHS	The Subjective Happiness Scale	Lyubomirsky and Lepper [95]
Life Satisfaction	SWLS	Satisfaction with Life Scale	Diener et al. [96]
Wellbeing	The PERMA Profiler	Positive Emotions, Engagement, Relationships, Meaning, and Accomplishments Profiler	Butler and Kern [97]
	PGWB	Psychological General Well-Being	Dupuy [98]
	WEMWBS	The Warwick Edinburgh Mental Wellbeing Scale	Stewart-Brown et al. [99]
	SCWBS	The Stirling Children’s Wellbeing Scale	Liddle and Carter [100]
Quality of Life	Q-LES-Q-SF	Quality of Life Enjoyment and Satisfaction Questionnaire–Short Form	Endicott et al. [101]
	WHOQOL-BREF	World Health Organization Quality of Life Assessment Brief Version	Whoqol Group [102]
Other	BASC-2	Behaviour Assessment System for Children, Second Edition	Reynolds and Kamphaus [103]
	BPNS	The Basic Psychological Need Satisfaction Scale	Deci and Ryan [104]
	BRCS	Brief Resilience Coping Scale	Sinclair and Wallston [105]
	BRS	Brief Resilience Scale	Smith et al. [106]
	EWL	Eigenschaftswörterliste (*List of Adjectives*)	Janke and Debus [107]
	ERC	Emotion Regulation Checklist	Shields and Cicchetti [108]
	FACT-G	Functional Assessment of Cancer Therapy-General scale Version 4;	Cella et al. [109]
	IES-R	Impact of Event Scale-Revised	Maercker and Schützwohl [110]
	IPIP	International Personality Item Pool	Goldberg [111]
	LOT-R	The Revised Life Orientation Test	Scheier et al. [112]
	MBI	Maslach Burnout Inventory	Maslach and Jackson [113]
	MDBF	Mehrdimensionalen Befindlichkeitsfragebogen (*Multidimensional Mood Questionnaire*)	Steyer et al. [114]
	RS-11	Resilience Scale-11	Kocalevent et al. [115]
	RSE	Rosenberg Self-Esteem Scale	Rosenberg et al. [116]
	PANAS	Positive and Negative Affect Schedule	Watson et al. [117]
	POMS-2	Profile of Mood States 2nd Edition	Heuchert and McNair [118]
	PSDQ	Physical Self-Description Questionnaire	Marsh et al. [119]
	SCS	The Social-Connectedness Scale	Lee and Robbins [120]
	SPANE	The Scale of Positive and Negative Experience	Diener et al. [121]
	STAXI	State-Trait Anger Expression Inventory	Spielberger [122]
	SDQ	Strengths and Difficulties Questionnaire	Goodman [123]
	SBQ-R	The Suicidal Behaviours Questionnaire-Revised	Osman et al. [124]
	RGCMS	Revised Geriatric Centre Morale Scale	Lawton [125]

**Table 2:** Full results table reporting demographics and relationship between attachment and mental health outcomes for included studies.

Author	Sample Size	Gender	Age	Country	Population	Pet Type	Attachment Measure	Mental Health Outcome/s	Mental Health Measure	Findings
**Studies finding higher attachment associated with better mental health on all measured variables**										
Black [126]	293	45.9% men 54.1% women	*M* = 15.8 years	USA	Adolescents	Dogs, cats, horses, other	CABS	Loneliness	UCLA LS	Higher pet attachment associated with lower loneliness
Douglas et al. [127]	187	38% men 61% women 1% transgender	*M* = 18.89	USA	College	Dogs, cats, other	LAPS; PAQ **	Suicide risk	SBQ-R;	Higher attachment associated with lower suicide risk.
Garrity et al. [128]	1232 (408 owners)	42.4% men 57.6% women	Majority (69.4%) aged 65–75 years *	USA	Elderly	Dogs, cats, other	6 items measure created for study	Depression	CES-D	Higher pet attachment associated with lower depression.
Hawkins et al. [129]	77	43% boys 57% girls	*M* = 10 years	Various	Children	Dogs	CENSHARE PAS	Emotional and social problems; emotional regulation	SDQ (parent-report); ERC (parent report)	Higher attachment associated with better emotional regulation and lower emotional and social problems.
Hawkins et al. [130]	77	43% boys 57% girls	*M* = 10 years	Various	Children	Dogs	CENSHARE PAS	Wellbeing; happiness; loneliness; social dissatisfaction; quality of life	SCWBS; SHS; The Children’s Loneliness and Social Dissatisfaction Scale [131], and KIDSCREEN-10 [132]	Higher attachment associated with higher scores for wellbeing, happiness, loneliness, social dissatisfaction and quality of life.
Luhmann and Kalitzki [133]	631	5.9%men 94.1% women	*M* = 27.8	Germany	General population	Dogs, cats, horses	CCAS	Life satisfaction; positive mood; need satisfaction; positive and negative affect; purpose and meaning in life	SWLS; Need Satisfaction Scale [134]; MDBF; PANAS; Two items adapted for purpose of life	Higher attachment associated with higher life satisfaction, more positive mood, more purpose, higher needs. satisfaction, more positive emotions and less negative emotions.
Marsa-Sambola et al. [60]	7159	44.8% boys 55.2% girls	*M* = 13.66	England and Scotland	Children	Not reported	SAPS	Wellbeing and life satisfaction	KIDSCREEN 10 and Single-item measure of Life Satisfaction [135]	Higher attachment associated with higher wellbeing and life satisfaction.
Marsa-Sambola et al. [136]	2262	46% boys 54% girls	Boys *M* = 13.02; Girls *M* = 13.50	Scotland	Children	Dogs and cats	SAPS	Wellbeing	KIDSCREEN 10	Higher attachment associated with higher wellbeing.
Ory and Goldberg [137]	1073	100% women	Range 65–75	USA	Elderly women	Dogs and cats	5-point scale from not at all to very attached	Happiness	Single item from Bradburn [138]	Those who were not very attached had lower happiness than those who were very attached (and those without pets).
Quan et al. [139]	407	47.2% men 52.8% women	*M* = 33	Korea	Tourists	Not reported	8 items adapted from Vada et al. [140]	Life satisfaction and stress	SWLS; Life stress adapted from Jiyeong et al. [141]	Higher pet attachment associated with higher life satisfaction and lower life stress.
Ribera et al. [142]	136	35.3%boys 64.7% girls	*M* = 9.01	Italy	Children	Dogs	DAQ; Archer and Ireland [55]	Emotional and social problems;	SDQ (parent-report)	Higher attachment predicted fewer child adjustment problems in a regression (was not significant in correlation).
Sung and Han [143]	263	36.9% men 63.1% women	Majority in 30 s	Korea	General population	Dogs	LAPS	Quality of life	WHOQOL-BREF	Higher attachment associated with better quality of life.
Tan et al. [144]	534 (431 owners)	20.4% men 79.6% women	*Mdn* = 29	Singapore	COVID-19	Dogs, cats, other	Questions adapted from PAQ and CENSHAREPAS	Emotional wellbeing	RAND 36-item Health Survey [145]	Higher attachment associated with higher emotional wellbeing.
Wen Li et al. [146]	160 (80 owners)	31.2% men 68.8% women	Categorical	Malaysia	General population	Dogs, cats, other	LAPS	Mental health; stress	SF-12; Perceived Stress Scale	Higher attachment associated with better mental health and lower stress.
Wong et al. [147]	275	32.4% men 67.6% women	Categorical	Taiwan	Children	Dogs, cats, other	8 questions from Stallones et al. [148]	Life satisfaction	Adapted from Diener et al. [96]	Higher pet attachment associated with higher life satisfaction.
**Studies finding higher attachment associated with better mental health on some variables, and no relationship with mental health on other variables**										
Budge et al. [149]	176	32% men 68% women	*M* = 42	New Zealand	General population	Dogs and cats	CENSHARE PAS	General mental health and wellbeing	MHI	Higher attachment associated with higher positive affect and wellbeing, but no relationship with mental health, psychological distress, emotional instability, depression or anxiety.
Israr et al. [150]	100	46% men 54% women	*M* = 23.71	Pakistan	General population	Not reported	PALS	Depression and anxiety	CES-D; BAI	Higher regulation and personal growth subscale associated with lower depression and anxiety. Love subscale not associated with either.
Muldoon et al. [151]	6700 (4817 owners)	48.7% boys 50.74% girls	11–15 years	Scotland	Children	Dogs, cats, other	SAPS	Wellbeing and quality of life	KIDSCREEN 10; 0–10 rating Quality of Life; GHQ-12; 1–4 rating of happiness	Higher attachment associated with higher QOL, wellbeing, and self-rated happiness, but no association with GHQ score for dog owners.
Namekata and Yamamoto [152]	180 (92% owners)	31.1% men 68.9% women	*M* = 19.4	Japan	University students during COVID-19	Dogs, cats, other	CAAS	Mood states	POMS2	Higher attachment associated with better overall mood and vigour, lower confusion and fatigue, but not associated with any of the other four mood subscales.
Paul and Serpell [153]	27	51.8% boys 48.2% girls	*M* = 9.70	UK	Children	Dogs	Rated on a visual analogue scale	Wellbeing	Continuous scale for how much the child feels various emotions (e.g., worried, lonely).	Higher attachment associated with more confidence and less tearfulness but not with the other four emotional outcomes.
Schwarzmueller-Erber et al. [154]	124	23.4% men 76.6% women	*M* = 56.94	Austria	Older adults	Dogs and horses	PALS	Wellbeing	FAHW 12; and 28 items created by study authors to measure wellbeing during and after walking dog or riding horse	Higher levels of overall pet attachment, and the love, regulation, and personal growth subscales of attachment were associated with higher social and psychological wellbeing. No relationship with attachment and general physical, psychological or social wellbeing.
Silva et al. [155]	106 (64 owners)	100% women	*Mdn* = 44 years	Portugal	People with Fibromyalgia	Dogs	MDORS	Anxiety and depression	The Portuguese version of the Hospital Anxiety and Depression Scale	Higher emotional closeness and dog-owner interaction, and lower perceived costs, associated with lower depression, adjusted for pain intensity and perceived social support. No variables were associated with anxiety.
Triebenbacher [156]	436 (385 owners)	53.2% boys 46.8 girls	Elementary *M* = 11; Middle *M* = 14; High school *M* = 16	USA	Children	Dogs, cats, other	CABS	Self-esteem	RSE	Higher attachment associated with higher self-esteem for the total sample and high schoolers, but no significant relationship for elementary and middle schoolers.
Wu et al. [157]	288	38.9% men 61.1% women	Majority aged 18 to 30	Hong Kong	General Population	Dogs, cats, other	CABS	Stress	PSS-10	Higher overall attachment score, emotional bond and caretaking subscales associated with lower levels of stress. Physical proximity subscale not related to stress.
Zebrowska et al. [158]	215	100% women	*M* = 60.8	USA	Nurses	Dogs, cats, other	6 questions from LAPS	Depression and anxiety	CES-D; K6; CCI; GAD-7	Higher pet attachment predicted lower depression and generalised anxiety, but not phobic anxiety.
**Studies finding attachment not related to mental health on any measured variables**										
Akiyama et al. [159]	108 (51 owners)	100% women	*M* = 57.4	USA	Widows	Dogs, cats, other	Katcher’s ten-item index of attachment to pets [160]	Depression	BDI	No relationship between attachment and depression.
Anderson [161]	34 (26 owners)	79.3% men 14.7% women *	Categorical	USA	Veterans with PTSD	Dogs	LAPS	Stress	PSS	No relationship between attachment and stress.
Angulo [162]	1872 (1110 owners)	100% men	*Mdn =* 38 *	USA	HIV patients	Dogs, cats, bird, other	CABS	Depression	CES-D	No relationship between attachment and depression.
Blanton [163]	135 (63 owners)	30.4% men 69.6% women *	Categorical	USA	University students	Not reported	CABS	Stress	PSS	No relationship between attachment and stress.
Bradshaw-Scott [164]	51	73% men 27% women	Categorical	USA	Veterans	Dogs	OPRS	Stress and quality of life	PSS; Q-LES-Q-SF	No relationship between attachment and stress or quality of life.
Branson et al. [165]	88 (48 owners)	29% men 71 % women	*M* = 74.35 years	USA	Elderly	Dogs, cats, other	Single-item attachment measure	Depression	GDS	No relationship between attachment and depression.
Branson et al. [166]	96 (41 owners)	24% men 76% women	*M* = 79.62 years	USA	Elderly	Cats	LAPS	Depression, stress, and loneliness	PSS, UCLA, GDS	No relationship between attachment and depression, stress or loneliness scores.
Carlisle et al. [167]	764 (626 owners)	9.8% men 90.2% women *	*M* = 44.9 *	USA	Parents with ASD children	Dogs, cats, other	LAPS	Stress	Parental SS	No relationship between parent attachment to pets and stress.
El-Alayli et al. [168]	70	33% men 67% women	*M* = 21	USA	University students	Dogs, cats, other	PAS (1996); CABS; 7-item Equal Family Member Status subscale of PRS	Satisfaction with life; positive and negative affect; happiness	SWLS; PANAS; SHS	No relationship between attachment and wellbeing.
Hartwig and Signal [169]	283	15.2% men 84.8% women	*M* = 16.1	Australia	Adolescents	Dogs, cats, other	CABS	Loneliness	UCLA LS v-3	No relationship between attachment and loneliness.
Howe [170]	81	24.7% men 75.3% women	*M* = 70.2	USA	Elderly	Dogs and cats	LAPS	Loneliness	ABLS	No relationship between attachment and loneliness.
Ingram and Cohen-Filipic [171]	122	5% men 95% women	*M* = 47.84	USA	Cancer patients	Dogs	LAPS	Depression, positive affect, quality of life	CES-D, The CES-D Positive affect subscale, FACT-G	No relationship between attachment and mental health.
Joseph et al. [172]	244 (122 owners)	46.7% men 53.3% women	Categorical	India	General population	Dogs and cats	LAPS	Stress	PSS	No relationship between attachment and stress.
Koontz [173]	202 (115 owners)	100% women	Median age 29.5 *	USA	Single mothers	Not reported	CCAS	Stress	PSS	No relationship between attachment and stress.
Kopser [174]	112	10.7% men 89.3% women	18+	USA	University students during COVID-19	Dogs, cats, other	LAPS	Stress and depression	PSS, PSQ; CSSQ; CES-D	No relationship between attachment and any outcomes.
Lewis et al. [43]	282 (144 owners)	19.5% men 80% women 0.5% other	Majority under 30	New Zealand	University students	Dogs, cats, other	6 items from Garrity et al. [128]	Quality of life	WHOQOLBREF	No relationship between attachment and quality of life.
Miller and Lago [175]	53	100% women	*M* = 73	USA	Elderly women	Dogs, cats, other	Pet Relationship Scale	Depression	GDS	No relationship between attachment to pets and depression.
Mueller et al. [176]	357 (195 owners)	34% men 65% women 1% other	longitudinal	USA	Adolescents during COVID-19	Dogs, cats, other	Network of Relation-ships Inventory-Pet (NRI-Pet)	Loneliness	3 items from Hughes et al. [177]	Attachment to pets did not predict loneliness.
Netting et al. [178]	75	17.3% men 82.7% women	*M* = 43.5	USA	General population	Dogs	LAPS	General mental health	SF-12	No relationship between attachment and mental health
Quinn [179]	305	38.6% men 61.3% women	*M* = 42.6	USA	General population	Dogs, cats, horses	CABS	Depression and anxiety	DASS	No relationship between attachment and depression or anxiety.
Raina et al. [180]	1054 at T1 (281 owners), 995 at T2 (245 owners)	48% men 52% women (at T2)	*M* = 73	Canada	Elderly	Dogs and cats	LAPS	Wellbeing	Created for this study	No relationship between and changes in psychological wellbeing.
Reddig [181]	51	35% boys 65% girls	*M =* 14.6	USA	Adolescents	Dogs, cats, other	LAPS; CABS	Internalizing problems, inattention/hyper-activity and personal adjustment	BASC-2	No relationship between attachment with pet and mental health outcomes.
Shoesmith et al. [182]	170 (81 owners)	52.4% men 52.4% women 1.2% other *	*M* = 52.19 *	UK	Participants with severe mental illness (psychotic disorders, bipolar etc.)	Dogs, cats, other	CCAS	Wellbeing, depression, anxiety	Four questions taken from Office for National Statistics Health and Lifestyle Survey [183]; PHQ-2; GAD-2	No relationship between attachment and mental health or wellbeing.
Smith [184]	76 (38 owners)	50% men 50% women	Men *M* = 51.5; Women *M* = 49.36	USA	Married couples	Dogs	LAPS	Stress	PSS	No relationship between attachment and stress.
Smolkovic et al. [185]	365	9.6% men 90.41% women	*M* = 28.4	Slovenia		Dogs and cats	OPRS	Loneliness	DLS	No relationship between attachment and loneliness.
Sobering [186]	219 (129 owners)	66.5% men 32.9% women 0.5% other	*M* = 67.3	USA	Elderly	Dogs, cats, other	LAPS	Wellbeing	The PERMA Profiler	No relationship between attachment and wellbeing.
Stickle [187]	352	11.6% men 88.4% women	Categorical	Canada		Dogs, cats, other	CABS plus 2 items created for this study	Resilience	BRCS	No relationship between attachment and resilience.
Turner et al. [188]	630	Not reported	Reported separately for each group of participants (*M* range from 45.3–52.8)	Switzerland	Couples and single people	Cats	LAPS; CABS	Mood states	EWL	No relationship between attachment and mood.
Wan et al. [189]	187	Not reported	*M* = 37	USA	COVID-19	Not reported	CCAS	Stress, depression, emotional exhaustion	Stress measured with four-item scale [190]; Emotional exhaustion with nine-item scale [191]; Depression with eight-item scale [192]	No direct relationship between attachment and the variables.
Watson and Weinstein [193]	84 (42 owners)	100% women	*M* = 38.9	USA	American Medical Association employees	Dogs and cats	8 questions from Stallones et al. [148]	Depression, anxiety and anger	BDI; STAI; STAXI	No relationship between attachment and mental health.
Winefield et al. [52]	314 (179 owners)	41.5% men 58.5% women	*M* = 71.1	Australia	Elderly	Dogs, cats, other	OPRS	Quality of life	SF-36 Health Survey	No relationship between attachment and mental health.
Wu [194]	196	25% men 75% women	Range 18–73	USA	University and community sample	Dogs, cats, other	Items taken from LAPS, PAQ, and CABS to form 5 new factors of attachment: Pet Provisions, Emotional Bond, Physical Proximity, Personal Growth, and Pet Care	Dissociation	DES	No relationship between attachment and mental health.
Zasloff and Kidd [195]	148 (59 owners)	100% women	*M* = 28.4	USA	Single women	Dogs and cats	PRS	Loneliness	UCLA-LS	No relationship between attachment and loneliness.
**Studies finding higher attachment associated with worse mental health on all measured variables**										
Barlow et al. [196]	83	24.3% men 75.7% women	Group *M* ranged from 19.26–35.35	USA	General population	Not reported	Pet Attachment and Life Impact Scale	Dissociation	DES	Participants with high dissociative symptoms and dissociative identity disorder diagnosis had higher attachment than those with low dissociative symptoms.
Brown and Katcher [197]	305	23% men 77% women	*M* = 20	USA	College students and vet technicians	Not reported	8 questions from Stallones et al. [148]	Dissociation	DES	Higher pet attachment associated with higher overall dissociation and all subscales.
Brown and Katcher [198]	113	All female	*M* = 23.29	USA	Veterinary technicians	Not reported	8 questions from Stallones et al. [148]	Dissociation	DES	Higher pet attachment associated with higher overall dissociation and all subscales.
Burnett [199]	191	Not Available	Not Available	USA	General population	Dogs and cats	LAPS	Depression	BDI II	Participants scoring in the highest 1/3 for depressive symptoms had higher attachment than those who the lowest 1/3.
Dowsett et al. [200]	313	11% men 89% women	*M* = 41.89	Online (country not specified)	General population	Dogs and cats	LAPS People substituting subscale	Separation anxiety (human and pets)	SA-10	People substituting associated with separation anxiety from humans and pets, but effect disappeared when analysing cat owners and human separation anxiety.
Harp [201]	77	29.9% men 70.1% women	*M* = 77.8	USA	Elderly	Dogs	LAPS	Depression, anxiety and loneliness	GDS-15; GAS-10; UCLA LS-v3	Higher attachment associated with higher loneliness, depression and anxiety.
Hou et al. [202]	547	45% men 55% women	*M* = 19.82	China	University students	Dogs, cats, others	LAPS	Loneliness	UCLA LS-v3	Higher attachment to pets associated with higher loneliness.
Hutton [203]	128 (77 owners)	92.2% men 7.8% women	*M* = 47.9	Australia	HIV patients	Dogs, cats, other	LAPS	Wellbeing	Emotional Wellbeing/Living with HIV subscale of Revised Functional Assessment of HIV Infection quality of life instrument Peterman et al. [204]	Higher attachment associated with lower wellbeing.
Keil [205]	275	31% men 69% women	*M* = 71	USA	Elderly	Cats, dogs, other	Not described	Stress and loneliness	RGCMS	Higher attachment associated with higher stress and loneliness.
Krause-Parello and Gulick [206]	191	16.8% men 83.2% women	*M* = 71	USA	Elderly	Dogs and cats	PAS (1988)	Loneliness	UCLA LS	Higher attachment associated with higher loneliness.
Krause-Parello [207]	159	100% women	*M* = 71	USA	Elderly women	Dogs and cats	PAS (1988)	Depression and loneliness	PGWB Depressed Mood Subscale; UCLA LS	Higher attachment associated with higher depression and loneliness.
Lass-Hennemann et al. [208]	580 (180 owners)	59.48% men 40.52% women	*M* = 38.19	Germany	High risk professions (police, firefighters, medical professionals)	Dogs and cats	LAPS	Resilience, general mental health, PTSD, burnout	RS-11; BSI; IES-R; MBI	Higher attachment to pets associated with poorer mental health, higher rates of PTSD and burnout.
Lass-Hennemann et al. [209]	610	7.05% men 92.79% women 0.16% non-binary	*M* = 33.12	Germany	General Population	Dogs	LAPS	General mental health	BSI	Higher attachment associated with poorer mental health.
McDonald et al. [210]	1942	7% men 89.8% women 3.4% other	*M* = 39.68	USA	COVID-19	Dogs and cats	LAPS	General mental health	8 of the 9 BSI subscales	Higher attachment associated with poorer mental health.
Miltiades and Shearer [211]	117	44% men/56% women	*M* = 68.42	USA	Elderly	Dogs	LAPS	Depression	CES-D	Higher attachment associated with higher depression.
Northrope et al. [212]	607	49.9% men 47.4% women 2.7% other	*M* = 32.1	Various	General population	Dogs	LAPS	General mental health	BSI	Higher attachment associated with worse mental health.
Peacock et al. [23]	150	17.4% men 82.6% women	*M* = 48.5	Australia	General population	Dogs, cats, other	CENSHARE PAS and OPRS combined to create an overall attachment score	General mental health	BSI-18	Higher attachment associated with worse mental health.
Pezzini [213]	304	41% men 44% women 15% not reported	*M* = 58	USA	General population	Dogs, cats, other	OPRS; PAQ **	Stress	PSS	Higher attachment associated with higher stress.
Rohlf et al. [214]	895	5.9% men 92.4% women 1.7% other	*M* = 42.25	Various	COVID-19	Dogs, cats, other	C/DORS Emotional Closeness subscale	Depression, anxiety, stress	DASS-21	Higher attachment associated with higher depression, anxiety and stress.
Tomich et al. [215]	423	22.5% men 77.5% women	*M* = 22.23	USA	University students	Dogs, cats, other	OPRS	Wellbeing	SF-12	Higher attachment associated with worse wellbeing.
Trautann [216]	169	Not reported	Median 45–54	USA	General population	Birds	LAPS	Loneliness	UCLA v-3	Higher attachment associated with higher loneliness.
Zoanetti et al. [217]	845	67% men 33% women	Median age range 45–54	Australia	Veterans	Dogs and cats	LAPS	General mental health	The Veterans RAND 12 Item Health Survey-mental health summary scale (MCS12)	Higher attachment associated with worse mental health.
**Studies finding higher attachment associated with worse mental health on some variables, and no relationship with mental health on other variables**										
Allen and Hogg [218]	639	19.2% men 80.7% women	*M* = 41	Australia	General population	Dogs	Emotional Closeness subscale of the MDORS	Positive and negative affect, loneliness	SPANE; SELSA-S;	Higher attachment associated with higher family-loneliness and romantic-loneliness. No relationship between attachment and social-loneliness or affect.
Antonacopoulos and Pychyl [219]	132 (66 owners)	32.2% men 67.7% women	*M* = 37.56	Canada	General population	Dogs and cats	LAPS	Depression and loneliness	CES-D; UCLA LS v-3	Attachment did not predict depression or loneliness. High attachment associated with higher depression and loneliness for those with low social support.
Atherton et al. [220]	735 (639 owners)	Autism Spectrum Disorder sample: 54% men 46% women * Neurotypical sample: 30% men 70% women *	Autism Spectrum Disorder sample: *M* = 28.64 * Neurotypical sample: *M* = 33.91 *	Various	Autistic spectrum disorder and neurotypicals	Dogs, cats, other	LAPS	Anxiety, life satisfaction, loneliness	LSAS; SWLS; UCLA-LS	For those with Autism Spectrum Disorder, LAPS General Attachment & People Substituting subscales score associated with of anxiety and loneliness. Animal rights subscale associated with anxiety only. No attachment variables associated with life satisfaction for either group. No relationship between attachment and outcome variables for neurotypicals.
Barklam and Felisberti [221]	495 (344 owners)	Study 1 22 men 78% women Study 2 25% men 75% women	Study 1 mean age 31.34, Study 2 mean age 27.63	Various	COVID-19	Dogs, cats, other	LAPS	Loneliness, wellbeing, resilience, optimism, need satisfaction	Single-item measure for loneliness; BRS LOT-R; BPNS	Study 1 People Substituting subscale associated with lower resilience and optimism. Animal welfare subscale associated with lower optimism. Study 2 Higher Total attachment associated with lower resilience. People Substituting subscale with higher levels of negative affect, lower levels of affect balance and resilience
Bennetts et al. [222]	1034	22% men 78% women	*M* parent age = 43.0; Child age = 0–17	Australia	COVID-19	Dogs and cats	CDORS Emotional Closeness subscale and subset of items from the CENSHARE PAS	Parent wellbeing and child anxiety	K6 and Spence Child Anxiety measure [223]	Higher child pet attachment associated with higher child anxiety. Higher parent–pet attachment associated with COVID-19 worry only. Parent Emotional Closeness associated with higher parental psychological distress.
Carr and Pendry [224]	145	16% men 84% women	*M* = 18.51	USA	College students	Dogs, cats, other	LAPS	Separation anxiety from pet; history of depression, anxiety, PTSD and self-harm	Adapted version of SA-10; self-reported history of mental illness	Higher overall attachment associated with higher separation anxiety and history of depression and anxiety and self-harm. All LAPS subscales associated with history of depression and anxiety. Higher General Attachment and People Substituting subscales associated with a history self-harm. Attachment not associated with PTSD.
Chopik et al. [225]	767 (424 owners)	15.3% men 81.7% women 3% other	*M* = 35.17	Various	COVID-19	Dogs, cats, other	2 items asking about level of comfort and connection with pet	Wellbeing; purpose; positive and negative affect; stress; loneliness; depression	Wellbeing single items [96]; Purpose single item [226]; Positive and negative affect four items each for from PANAS; Stress two items from PANAS; loneliness with two items [177]; Stress two items [227]	Higher attachment associated with higher stress. No relationship with other variables.
Gerber [228]	276 (211 owners)	35.9% men 64.9% women *	*M* = 22.7 *	South Africa	University students	Dogs, cats, other	LAPS	Quality of life	WHOQOL-BREF	Higher scores on the LAPS Animal Welfare subscale associated with lower overall quality of life and psychological health subscale. LAPS overall score and General Attachment and People Substituting subscales not related to overall quality of life or psychological health.
Hall et al. [229]	37 (17 in analysis)	80–85.7% women	Not reported	UK	Parents of children with Autism Spectrum Disorder	Dogs	LAPS	Parent stress	PSI-SF	Higher attachment associated with higher ratings of child being difficult, otherwise not related to parental stress.
Harlinger [230]	323	72.8% women	*M* = 40.44	USA	General population	Dogs	LAPS	Stress	PSS-10	Higher attachment associated with higher stress levels after an intervention asking them to think about playing with their dog, but not stress levels before the intervention.
le Roux and Wright [231]	3329 (3108 owners)	14% men 86% women *	*M* = 41 *	South Africa	General population	Dogs, cats, other	CCAS	Stress; life satisfaction	PSS; SWLS	Higher attachment associated with higher stress. No relationship between attachment and life satisfaction.
Matijczak et al. [232]	134	7.5% men 43.3% women 49.2% other	*M* = 19.31	USA	LGBQT	Dogs, cats,	CCAS	Depression; anxiety	BSI depression and anxiety subscales	Higher attachment associated with higher anxiety. No relationship with depression.
Matijczak et al. [233]	163	8.6% men 42.3% women 49.1% other	*M* = 19.31	USA	LGBTQ	Dogs and cats	PALS	Depression	BSI depression subscale	Love subscale of PALS only associated with depressive symptoms.
Oliva and Johnston [59]	526	30.6% men 68.1% women 1.3% other	*M* = 44.1	Various	COVID-19	Dogs and cats	POCS	Loneliness	The UCLA-LS V3	Connectedness with Other associated with higher loneliness. Owner–Pet Connection not associated with loneliness.
Platto et al. [234]	261	26% men 74% women	Categorical	China	COVID-19	Dogs and cats	CABS	Stress	PSS-10: anger/stress, lack of control and confidence subscales	Perceived anger/stress and lack of control associated with more negative bond. Perceived confidence associated with less negative bond. Lack of control associated with more proximity with pet. No relationship between other subscales.
Pranschke [235]	Study 1: 103; Study 2; 164 (85 owners) Study 3; 50	Study 1 24.3% men 72.8% women 2.9% other Study 2 38.4% men 60.3% women 0.01% other * Study 3 40% men 60% women	Study 1 *M* = 38.07; Study 2 *M* = 47.14 *; Study 3 *M* = 41.54	Canada	General population	Dogs, cats, other	LAPS	Depression; quality of life, loneliness	CESD-10; Quality of life-1 item from Revicki et al. [236]; Loneliness-3 items from Hughes et al. [177]	Higher scores on People Substituting associated with higher depression, but no other relationship between attachment and mental health in study 1 and 2. In study 3, People Substituting associated with higher loneliness, but not depression or quality of life.
Ratschen et al. [237]	5926 (5323 owners)	20.6% men 78.6% women 0.6% other *	Categorical	UK	COVID-19	Dogs, cats, others	CCAS	Mental health; wellbeing; loneliness	WEMWBS, the mental health subscale of the SF-36; 3-item version of the UCLA LS	Higher attachment associated with poorer mental health pre-lockdown, but not since lockdown.
Reevy and Delgado [238]	1239	12.5% men 87.5% women	*M* = 41	Various	General population	Cats	LAPS; PAQ **	6 facets of neuroticism-anger, anxiety, depression, immoderation; self-consciousness, and vulnerability, and overall neuroticism	Neuroticism facets from the IPIP	Higher attachment associated with higher overall neuroticism, anger, anxiety, depression and vulnerability
Stallones et al. [148]	1300 (598 owners)	52.1% men 47.9% women *	Categorical	USA	General population	Not reported	8 questions created for this study	Depression	CES-D Scale	Higher attachment associated with higher depression in the 35–44 age group only.
Teo and Thomas [239]	498 (322 owners)	29% men 71% women	*M* = 24.19	Australia	Psychology students and members of the public	Dogs, cats, other	OPRS; PAQ **	Depression, anxiety, Stress; general mental health; quality of life	DASS-21; BSI; WHOQOL-BREF	Higher attachment associated with poorer psychological quality of life, but no other mental health variable.
Tomlinson et al. [240]	138	7.2% men 44.2% women 48.6% other	*M* = 19.33	USA	Sexual and gender minority	Dogs, cats, other	CCAS	General mental health	BSI	Higher attachment associated with higher anxiety, but no other aspect of mental health.
Wells et al. [241]	249 (146 owners)	17.1% men 82.9% women	Categorical	UK	COVID-19	Dogs and Cats	LAPS; CABS	Depression; positive experience; loneliness; stress	PHQ-9; SPANE-P; 3-item UCLA LS; PSS	Higher attachment associated with higher depression and loneliness, and lower positive experience, but unrelated to stress.
Wong et al. [242]	275	32.9% men 67.1% women	Majority of participants aged 21–30	Taiwan	General population	Dogs and cats	7 questions from Stallones et al. [148]	Life satisfaction; emotional exhaustion	SWLS; Emotional Exhaustion subscale of MBI	Higher attachment associated with higher emotional exhaustion. No relationship with life satisfaction.
Wright [243]	3329 (3108 owners)	13.51% men 85.48% women	*M* = 41	South Africa	General population	Dogs, cats, other	CCAS	Stress; life satisfaction	PSS-10; SWLS	Higher attachment associated with higher stress. No relationship with life satisfaction.
**Studies finding higher attachment associated with worse mental health on some variables, and better mental health on others**										
Ellis et al. [244]	1359	44.9% men 54% women 1.1% other	*M* = 40.7	Various	General population	Dogs and cats	LAPS	Depression; anxiety; loneliness; positive and negative affect	PHQ-9; GAD-7; PANAS; UCLA LS;	Higher attachment associated with higher depression, anxiety, loneliness, and positive affect, unrelated to negative affect.
Martos Martinez-Caja et al. [245]	6772 (6520 owners)	13.1% men 86.7% women 0.2% Other *	Categorical	Various	COVID-19	Dogs, cats, horses, other	CCAS	Loneliness; positive and negative affect	PANAS; self-rated loneliness	Higher attachment associated with higher positive and higher negative affect. No relationship with loneliness.
**Studies investigating attachment style to pets and mental health**										
Chan and Wong [246]	229 (108 owners)	29.6% men 70.4% women	Categorical	Hong Kong	General population	Dogs	Modified ECR	Mental health	GHQ-12	Higher pet attachment anxiety associated with poorer mental health. No relationship with pet attachment avoidance.
da Silva Roma [247]	401	10.7% male 88.4% women 0.9% non-binary	17–25	Canada	College students during COVID-19	Dogs	PAQ	Stress, loneliness; self-esteem; social connectedness	UCLA LS v-3; PSS; PSDQ; and SCS to create an overall wellbeing scale	Higher anxious and avoidant attachment associated with lower overall wellbeing.
Douglas et al. [127]	187	38% men 61% women 1% transgender	*M* = 18.89	USA	College	Dogs, cats other	LAPS; PAQ **	Suicide risk	SBQ-R	Higher anxious attachment style associated with higher suicidal tendencies.
Demeter [248]	52 (25 owners)	64% men 36% women	*M* = 67.6 years	Israel	Stroke patients	Dogs, cats, other	PAQ	Quality of life	WHOQOL-BREF	No relationship between attachment style and quality of life.
Langston [249]	561	13% men 85.7% women 1.2% other	*M* = 22.71	Mostly (89.9%) USA	College and general population	Dogs, cats, other	PAQ	Life satisfaction; positive and negative affect	PANAS	Higher avoidance was associated with lower positive affect only. Higher levels of anxiety were associated with lower subjective wellbeing and positive affect, and higher negative affect.
Lee [250]	384	4.2% men 93.5% women 2.3% other	*M* = 39.10	Various	General population	Dogs, cats, other	PAQ	Quality of life	WHOQOL-BREF	Higher anxiety associated with lower psychological quality of life. Avoidance was not.
Liupakorn [251]	208	24% men 75.5% women 0.5% trans	*M* = 34.74	USA	General population	Dogs	PAQ	Happiness; Life satisfaction	OHQ; SWLS	No differences between those who were classified as secure or insecure.
Luchesi et al. [252]	301	27.2% men 72.8% women	*M* = 38.67	Brazil	General population	Cats	PAQ	Life satisfaction	SWLS	No relationship between avoidant and anxious attachment and satisfaction with life.
Northrope et al. [212]	607	49.9% men 47.4% women 2.7% other	*M* = 32.1	Various	General population	Dogs	LAPS; PAQ **	General mental health	BSI	Higher anxious and avoidant attachment associated with worse mental health.
Pezzini [213]	304	41% men 44% women 15% not reported	*M* = 58	USA	General population	Dogs, cats, other	OPRS; PAQ **	Stress	PSS	Higher avoidant attachment associated with higher levels of stress. Higher levels of anxious attachment associated with higher levels of stress only when avoidance and LAPS score not controlled for.
Reevy and Delgado [238]	1239	12.5% men 87.5% women	*M* = 41	Various	General population	Cats	LAPS; PAQ **	6 facets of neurotic-ism: anger, anxiety, depression, immoderation; self-consciousness, and vulnerability, and overall neuroticism	Neuroticism facets from the IPIP	Higher anxiety associated with higher overall neuroticism, anger, anxiety, depression, vulnerability. immoderation and self-consciousness. Avoidance not associated with any of these.
Ståhl et al. [253]	2724	3.6% men 92.69% women 6.3% other	Modal age group 25–29	Finland	General population	Dogs and Cats	PAQ	Overall wellbeing based on life satisfaction, stress, wellbeing, anxiety, and depression	SWLS, PSS-10; WEMWBS; GAD-7; and CES-D combined to create a general wellbeing scale	Higher avoidant and anxious attachment associated with poorer wellbeing.
Teo and Thomas [239]	498 (322 owners)	29% men 71% women	*M* = 24.19	Australia	Psychology students and members of the public	Dogs, cats, other	OPRS; PAQ **	Depression, anxiety, stress; general mental health; Quality of life	DASS-21; BSI; WHOQOL-BREF	Pet attachment anxiety associated with higher rates of depression, anxiety stress, poorer overall mental health and poorer quality of life in the psychological and social domain, but not physical or environmental.
Zilcha-Mano et al. [34]	212	31.1% men 68.9% women	*M* = 25.4	Israel	General	Dogs and cats	PAQ	General mental health and wellbeing	MHI	Pet attachment anxiety associated with higher distress and lower wellbeing. Pet attachment avoidance not associated with either.

* This refers to the demographics of the total sample, rather than pet owners specifically. ** Studies marked with this measured attachment in terms of strength and style, with results for each of these reported in the appropriate section.

**Table 3:** Results of the quality assessment according to the relevant JBI criteria.

Author	Year	Were the Criteria for Inclusion in the Sample Clearly Defined?	Were the Study Subjects and the Setting Described in Detail?	Was the Exposure Measured in a Valid and Reliable Way?	Were the Outcomes Measured in a Valid and Reliable Way?	Was Appropriate Statistical Analysis Used?
Akiyama et al. [159]	1986	Yes	Yes	Unclear	Yes	Yes
Allen and Hogg [218]	2022	Yes	Yes	Yes	Yes	Yes
Anderson [161]	2018	Yes	Yes	Yes	Yes	Yes
Angulo [162]	1996	Yes	Yes	Yes	Yes	Yes
Antonacopoulos [219]	2010	Yes	Yes	Yes	Yes	Yes
Atherton et al. [220]	2023	Yes	Yes	Yes	Yes	Yes
Barklam and Felisberti [221]	2023	Yes	Yes	Yes	Unclear *	Yes
Barlow [196]	2012	Yes	No	Yes	Yes	Yes
Bennetts et al. [222]	2022	Yes	Yes	Yes	Yes	Yes
Black [126]	2012	Yes	Yes	Yes	Yes	Yes
Blanton [163]	2019	Yes	No	Yes	Yes	Yes
Bradshaw-Scott [164]	2017	Yes	Yes	Yes	Yes	Yes
Branson et al. [166]	2019	Yes	Yes	Yes	Yes	Yes
Branson et al. [165]	2016	Yes	Yes	No	Yes	Yes
Brown and Katcher [198]	2001	Yes	No	Yes	Yes	Yes
Brown and Katcher [197]	1997	Yes	No	Yes	Yes	Yes
Budge et al. [149]	1998	Yes	Yes	Yes	Yes	Yes
Burnett [199]	2009	Yes	No	Yes	Yes	Yes
Carlisle et al. [167]	2020	Yes	Yes	Yes	Yes	Yes
Carr and Pendry [224]	2021	Yes	Yes	Yes	Unclear	Yes
Chan and Wong [246]	2022	Yes	Yes	Yes	Yes	Yes
Chopik et al. [225]	2023	Yes	Yes	No	Unclear *	Yes
da Silva Roma [247]	2023	Yes	Yes	Yes	Yes	Yes
Demeter [248]	2022	Yes	Yes	Yes	Yes	Yes
Douglas et al. [127]	2023	Yes	Yes	Yes	Yes	Yes
Dowsett et al. [200]	2020	Yes	Yes	Yes	Yes	Yes
El-Alayli et al. [168]	2006	Yes	Yes	Unclear	Yes	Yes
Ellis et al. [244]	2024	Yes	Yes	Yes	Yes	Yes
Garrity et al. [128]	1989	Yes	No	Yes	Yes	Yes
Gerber [228]	2016	Yes	Yes	Yes	Yes	Yes
Hall et al. [229]	2016	Yes	Yes	Yes	Yes	Yes
Harlinger [230]	2017	Yes	Yes	Yes	Yes	Yes
Harp [201]	2024	Yes	Yes	Yes	Yes	Yes
Hartwig and Signal [169]	2020	Unclear	Yes	Yes	Yes	Yes
Hawkins et al. [129]	2022	Yes	Yes	Yes	Yes	Yes
Hawkins et al. [130]	2023	Yes	Yes	Yes	Yes	Yes
Hou et al. [202]	2021	Unclear	Yes	Yes	Yes	Yes
Howe [170]	1995	Yes	Yes	Yes	Yes	Yes
Hutton [203]	2014	Yes	Yes	Yes	Yes	Yes
Ingram and Cohen-Filipic [171]	2019	Yes	Yes	Yes	Yes	Yes
Israr et al. [150]	2022	Yes	No	Yes	Yes	Yes
Joseph et al. [172]	2019	Yes	Yes	Yes	Yes	Yes
Keil [205]	1998	Yes	Yes	Unclear	Yes	Yes
Koontz [173]	2009	Yes	Yes	Yes	Yes	Yes
Kopser [174]	2023	Yes	Yes	Yes	Yes	Yes
Krause-Parello and Gulick [206]	2013	Yes	Yes	Yes	Yes	Yes
Krause-Parello [207]	2012	Yes	Yes	Yes	Yes	Yes
Langston [249]	2014	Yes	Yes	Yes	Yes	Yes
Lass-Hennemann et al. [208]	2020	Yes	Yes	Yes	Yes	Yes
Lass-Hennemann et al. [209]	2022	Yes	Yes	Yes	Yes	Yes
Lee [250]	2021	Yes	Yes	Yes	Yes	Yes
le Roux and Wright [231]	2020	Yes	Yes	Yes	Yes	Yes
Lewis et al. [43]	2009	Yes	Yes	Yes	Yes	Unclear
Liupakorn [251]	2019	Yes	Yes	Yes	Yes	Unclear
Luchesi et al. [252]	2022	Yes	Yes	Yes	Yes	Yes
Luhmann and Kalitzki [133]	2018	Yes	Yes	Yes	Unclear *	Yes
Marsa-Sambola et al. [60]	2016	Yes	Unclear	Yes	Yes	Yes
Marsa-Sambola et al. [136]	2017	Yes	Yes	Yes	Yes	Yes
Martos Martinez-Caja et al. [245]	2022	Yes	Yes	Yes	Yes	Yes
Matijczak et al. [232]	2020	Yes	Yes	Yes	Yes	Yes
Matijczak et al. [233]	2022	Yes	Yes	Yes	Yes	Yes
McDonald et al. [210]	2021	Yes	Yes	Yes	Yes	Yes
Miller and Lago [175]	1990	Yes	Yes	Yes	Yes	Yes
Miltiades and Shearer [211]	2011	Yes	Yes	Yes	Yes	Yes
Mueller et al. [176]	2021	Yes	Yes	Yes	Yes	Yes
Muldoon et al. [151]	2019	Yes	Yes	Yes	Yes	Unclear
Namekata and Yamamoto [152]	2021	Yes	Yes	Yes	Yes	Yes
Netting et al. [178]	2013	Yes	Yes	Yes	Yes	Yes
Northrope et al. [212]	2024	Yes	Yes	Yes	Yes	Yes
Oliva and Johnston [59]	2022	Yes	Yes	Yes	Yes	Yes
Ory and Goldberg [137]	1983	Yes	Yes	Unclear	Unclear	Yes
Paul and Serpell [153]	1996	Yes	Yes	Unclear	Unclear	Yes
Peacock et al. [23]	2012	Yes	Yes	Yes	Yes	Yes
Pezzini [213]	2016	Yes	Yes	Yes	Yes	Yes
Platto et al. [234]	2022	Yes	Yes	Yes	Yes	Yes
Pranschke [235]	2019	Yes	Yes	Yes	Unclear *	Yes
Quan et al. [139]	2023	Yes	Unclear	Unclear	Unclear *	Unclear
Quinn [179]	2006	Yes	Yes	Yes	Yes	Yes
Raina [254]	1996	Yes	Yes	Yes	Yes	Unclear
Ratschen et al. [237]	2020	Yes	Yes	Yes	Yes	Yes
Reddig [181]	2019	Yes	Yes	Yes	Yes	Yes
Reevy and Delgado [238]	2020	Yes	Yes	Yes	Yes	Yes
Ribera et al. [142]	2023	Yes	Yes	Yes	Yes	Yes
Rohlf et al. [214]	2024	Yes	Yes	Yes	Yes	Yes
Schwarzmueller-Erber et al. [154]	2020	Yes	Yes	Yes	Yes	Yes
Shoesmith et al. [182]	2023	Yes	Yes	Yes	Unclear	Yes
Silva et al. [155]	2021	Yes	Yes	Yes	Yes	Yes
Smith [184]	2004	Yes	Yes	Yes	Yes	Yes
Smolkovic et al. [185]	2012	Yes	Yes	Yes	Yes	Yes
Sobering [186]	2023	Yes	Yes	Yes	Yes	Yes
Ståhl et al. [253]	2023	Yes	Yes	Yes	Yes	Yes
Stallones et al. [148]	1990	Yes	Unclear	Yes	Yes	Yes
Stickle [187]	2012	Yes	Yes	Yes	Yes	Yes
Sung and Han [143]	2023	Unclear	Yes	Yes	Yes	Yes
Tan et al. [144]	2021	Yes	Yes	Unclear	Yes	Yes
Teo and Thomas [239]	2019	Yes	Yes	Yes	Yes	Yes
Tomich et al. [215]	2024	Yes	Yes	Yes	Yes	Yes
Tomlinson et al. [240]	2021	Yes	Yes	Yes	Yes	Yes
Trautann [216]	2023	Yes	No	Yes	Yes	Yes
Triebenbacher [156]	1998	Yes	Yes	Yes	Yes	Yes
Turner et al. [188]	2003	Yes	Yes	Yes	Yes	Yes
Wan et al. [189]	2023	Yes	No	Yes	Yes	Yes
Watson and Weinstein [193]	1993	Yes	Yes	Yes	Yes	Yes
Wells et al. [241]	2022	Yes	Yes	Yes	Yes	Yes
Wen Li et al. [146]	2017	Yes	Yes	Yes	Yes	Yes
Winefield et al. [52]	2008	Yes	Yes	Yes	Yes	Yes
Wong et al. [147]	2023	Yes	Yes	Yes	Yes	Yes
Wong et al. [242]	2024	Yes	Yes	Unclear	Yes	Yes
Wright [243]	2018	Yes	Yes	Yes	Yes	Yes
Wu et al. [157]	2018	Yes	Yes	Yes	Yes	Yes
Wu [194]	2019	Yes	Yes	Yes	Yes	Yes
Zasloff and Kidd [195]	1994	Yes	Yes	Yes	Yes	Unclear
Zebrowska et al. [158]	2024	Yes	Yes	Unclear	Yes	Yes
Zilcha-Mano Zilcha-Mano et al. [34]	2011	Yes	Yes	Yes	Yes	Yes
Zoanetti et al. [217]	2023	Yes	Yes	Yes	Yes	Yes
% of studies that were Yes for quality assessment		97.44%	89.74%	85.47%	76.92%	78.63%

* At least one of the measures used could not be clearly confirmed as valid or reliable.

## Data Availability

Not applicable.
